# Epidemiology of positive bacterial cultures and the coverage gaps in multiplex PCR diagnostics: a single-center retrospective study

**DOI:** 10.1128/jcm.01600-24

**Published:** 2025-02-26

**Authors:** Vishwaratn Asthana, Rishi Chanderraj, J. Scott VanEpps, Robert P. Dickson

**Affiliations:** 1 Department of Internal Medicine, University of Michigan Division of Hospital Medicine 173243 , Ann Arbor, Michigan, USA; 2 Division of Infectious Diseases, Department of Internal Medicine, University of Michigan Medical School 12266 , Ann Arbor, Michigan, USA; 3 Medicine Service Infectious Diseases Section, Veterans Affairs (VA) Ann Arbor Healthcare System, Ann Arbor, Michigan, USA; 4 University of Michigan Weil Institute for Critical Research and Innovation 1259 , Ann Arbor, Michigan, USA; 5 University of Michigan Department of Emergency Medicine - Adult 613872 , Ann Arbor, Michigan, USA; 6 Department of Biomedical Engineering, University of Michigan 505527 , Ann Arbor, Michigan, USA; 7 University of Michigan Program in Macromolecular Science and Engineering 1259 , Ann Arbor, Michigan, USA; 8 University of Michigan Biointerfaces Institute 637750 , Ann Arbor, Michigan, USA; 9 Department of Internal Medicine, University of Michigan Medical School Division of Pulmonary and Critical Care Medicine 12266 , Ann Arbor, Michigan, USA; 10 Department of Microbiology and Immunology, University of Michigan Medical School 12266 , Ann Arbor, Michigan, USA; Universitat Münster, Münster, Germany

**Keywords:** bacterial incidence, clinical microbiology, multiplex PCR, diagnostics

## LETTER

Multiplex PCR panels have become essential tools in clinical microbiology, providing rapid detection of pathogens in blood, urine, and respiratory specimens. Yet by definition, targeted molecular assays detect a finite range of bacterial species, lacking the taxonomic breadth of untargeted techniques like cultivation and metagenomics. While, in recent years, multiplex panels have increased in their number of target species, current panels still are not sufficiently broad to encompass all clinically important pathogens. While prior studies have compared earlier (more taxonomically narrow) PCR assays in specific anatomic compartments (1), to our knowledge, no study has determined the relative coverage of contemporary PCR-based approaches as compared to culture-identified microbiota in a real-world setting.

To address this, we designed a retrospective observational study in which we compared the theoretical coverage of diagnostic multiplex PCR platforms with culture-identified pathogens in an academic clinical microbiology laboratory. We analyzed all positive blood, urine, and respiratory cultures between 2019 and 2023 (representing 29,264, 49,990, and 32,272 isolates, respectively) at Michigan Medicine—a large, Midwest, tertiary care academic center. Specimens were cultured according to clinical microbiology protocols, and isolates were identified using MALDI-TOF (Bruker) (2, 3). Repeat samples from the same patient were not excluded. Bacterial isolates were cultured from 9.2% of blood specimens, 32.0% of urine specimens, and 51.1% of respiratory specimens. The data were retrieved from the Clinical Microbiology laboratory information management system. The data were deidentified and aggregated, and so were IRB exempt. We excluded fungal and mycobacterial isolates given our focus on bacterial pathogens, the primary use for the assays in question. To determine the minimum number of targets needed to attain a given percentage of isolates, we calculated the cumulative fraction of all isolates (based on descending order of frequency).

**Fig 1:**
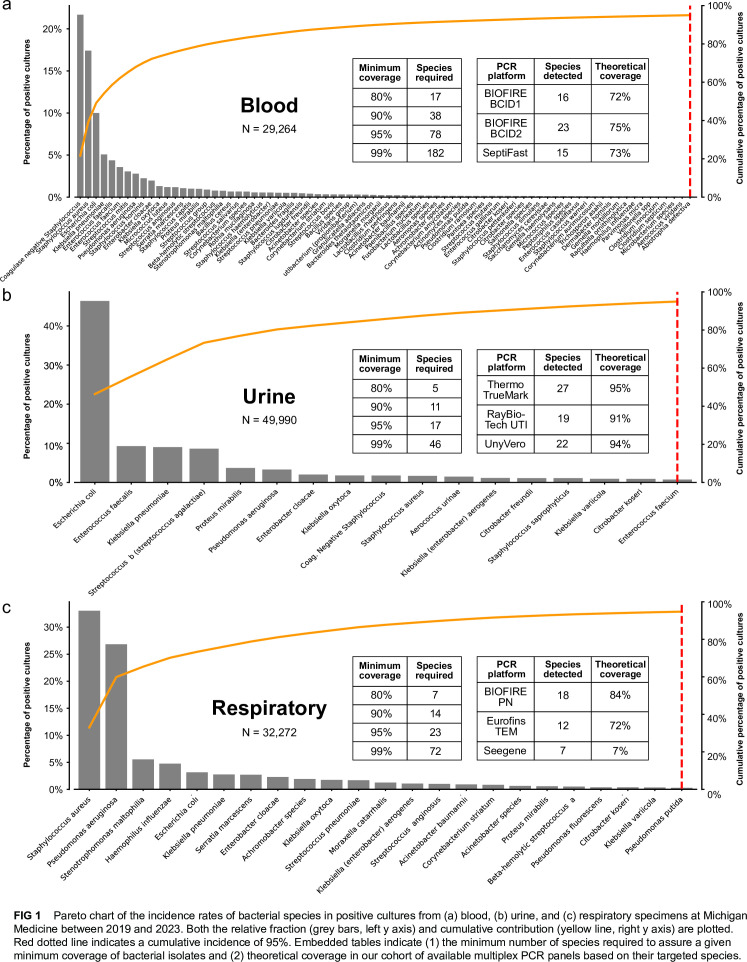
Pareto chart of the incidence rates of bacterial species in positive cultures from (a) blood, (b) urine, and (c) respiratory specimens at Michigan Medicine between 2019 and 2023. Both the relative fraction (gray bars, left y-axis) and cumulative contribution (yellow line, right y-axis) are plotted. The red dotted line indicates a cumulative incidence of 95%. Embedded tables indicate (1) the minimum number of species required to assure a given minimum coverage of bacterial isolates and (2) theoretical coverage in our cohort of available multiplex PCR panels based on their targeted species.

As shown in Fig. 1, for a targeted assay to attain 95% theoretical coverage of all positive bacterial isolates, multiplex panels would need to detect 78 bacterial species in blood specimens, 17 bacterial species in urine, and 23 species in respiratory specimens.

We next compared existing PCR-based assays in their theoretical coverage of real-world bacterial isolates. We identified relevant assays via a search of PubMed and recent review articles. As shown in the Pareto chart in Fig. 1, theoretical coverage was greatest among urinary assays (range 91%–95%) and lowest among blood assays (range 72%–75%).

Our results reveal that multiplex PCR platforms, even in their latest iterations, offer only incremental improvements in coverage compared to prior versions. For example, the BIOFIRE Blood Culture Identification 2 (BCID2) Panel theoretically identifies species in 75% of positive blood cultures, only 3% more than its predecessor, BCID1, albeit with improved species-level targeting (4–12).

Our study was limited by its reliance on single-center data and its focus on bacterial (rather than fungal and mycobacterial) pathogens. We did not include all existing multiplex platforms (including precursors to currently available assays). Yet, it is clear that existing multiplex PCR assays continue to have limited taxonomic coverage in real-world clinical microbiology settings, especially for blood and respiratory specimens. Our results suggest an inherent performance ceiling for multiplex panels and that further iterations and expansions will offer only diminishing returns. This issue is compounded by emerging multiplex assays attempting to do more with less, including coverage of non-bacterial pathogens and inference of antibiotic susceptibility. Due to inherent constraints in targeted PCR platforms (including the complexity of primer design, competition among targets for amplification, and the need for extensive optimization of reaction conditions), multiplex PCR panels may never attain sufficient taxonomic coverage to replace untargeted approaches given limits on the number of concurrent primers that can be multiplexed (13–15). These findings highlight the need for diagnostic systems that can adapt and expand to accommodate a broader spectrum of pathogens (16, 17).

Raw counts of bacterial identifications and specific pathogens covered by each multiplex assay are available in the Supplemental Material.

## AFTER EPUB

[This article was published on 26 February 2025 with errors in the text, figure, and supplemental file. The errors were corrected in the current version, posted on 28 May 2025.]

## SUPPLEMENTAL MATERIAL

Data S1 jcm.01600-24-s0001.csv Count of bacterial identifications from all positive blood cultures at UoM from 2019 to 2023.

Data S2 jcm.01600-24-s0002.csv Count of bacterial identifications from all positive sputum cultures at UoM from 2019 to 2023.

Data S3 jcm.01600-24-s0003.csv Count of bacterial identifications from all positive urine cultures at UoM from 2019 to 2023.

Data S4 jcm.01600-24-s0004.xlsx Bacteria covered by the multiplex assays discussed in this paper.

ASM does not own the copyrights to Supplemental Material that may be linked to, or accessed through, an article. The authors have granted ASM a non-exclusive, world-wide license to publish the Supplemental Material files. Please contact the corresponding author directly for reuse.
